# Effect of Oral Chinese Herbal Preparations Regulating Intestinal Flora on Lipid Metabolism Disorders in Patients: A Meta-Analysis of Controlled Clinical Studies

**DOI:** 10.3389/fsurg.2022.892438

**Published:** 2022-05-03

**Authors:** Wenqian Gong, Wuguang Zhang, Chunyang Chang

**Affiliations:** ^1^Department of Traditional Chinese Medicine, The Affiliated People's Hospital of Ningbo University, Ningbo, China; ^2^Oncology Department of Integrated Traditional Chinese and Western Medicine, The Affiliated People's Hospital of Ningbo University, Ningbo, China; ^3^Department of Emergency, Tongde Hospital of Zhejiang Province, Hangzhou, China

**Keywords:** intestinal flora, traditional Chinese medicine, lipid metabolism disorders, oral Chinese herbal, meta-analysis

## Abstract

**Background:**

Lipid metabolism disorders can damage human health, and the changes in human intestinal flora are closely related to lipid metabolism disorders. Traditional Chinese medicine (TCM) can play a role in regulating intestinal flora and balancing intestinal microecology. In this meta-analysis, the role of oral preparations of TCM that regulate intestinal flora, in the prevention and treatment of lipid metabolism disorders, was systematically evaluated.

**Methods:**

The databases CBM, Pubmed, Embase, CNKI, Wanfang, and Google Scholar were searched by rapid matching of keywords to obtain clinical controlled studies related to oral preparations of TCMs regulating intestinal flora. After screening and quality evaluation, meta-analysis was performed using Review Manager 5.3 software.

**Results:**

Total of 835 patients were enrolled in the 10 articles included in this study. Meta-analysis showed that TCM intervention could reduce the level of total cholesterol (TC) in patients with abnormal lipid metabolism [*mean difference (MD)* = −0.61, 95% confidence interval (95%CI) (−0.80, −0.42), *p* < 0.00001], reduce triacylglycerol (TG) level [*MD* = −0.46, 95%CI (−0.60, −0.33), *p* < 0.00001], increase high-density lipoprotein (HDL) level [*MD* = 0.25, 95%CI (0.17, 0.34), *p* < 0.00001], reduce the number of intestinal enterobacteria [*MD* = −0.64, 95%CI (−0.79, −0.49), *p* < 0.00001], reduce the number of enterococci [*MD* = −1.14, 95%CI (−1.66, −0.63), *p* < 0.00001], increase the number of intestinal lactobacillus [*MD* = 0.41, 95%CI (0.09, 0.74), *p* = 0.01], and increase the number of intestinal bifidobacteria [*MD* = 0.94, 95%CI (0.20, 1.68), *p* = 0.01].

**Conclusion:**

The application of oral preparations of TCMs that regulate intestinal flora, in the prevention and treatment of lipid metabolism disorders, can increase the colonization of beneficial bacteria in the intestine of patients, inhibit the growth of harmful bacteria, and restore the intestinal microecological balance, thus indirectly acting on the regulation of blood lipids in patients and contributing to the recovery of dyslipidemia.

## Introduction

Lipid metabolism disorders, also known as dyslipidemia, refer to the abnormal increase of one or more of the indicators of triacylglycerol, total cholesterol (TC), high-density lipoprotein (HDL), and low-density lipoprotein (LDL) in human serum (1). With the gradual improvement of people's living standards and changes in dietary structure in China, dyslipidemia has become one of the major diseases threatening the health of middle-aged and elderly people, and is the main cause of atherosclerosis (AS) and coronary heart disease (CHD), damaging the health of patients (2). Lipid metabolism disorders often emerge with diseases such as obesity, diabetes, and ovarian syndrome, causing complex effects on the human body (3). Therefore, it is clinically important to seek treatments that effectively regulate the disorders of lipid metabolism and prevent the occurrence of dyslipidemia. Some studies (4) have found that the changes in intestinal flora in human body are closely related to lipid metabolism disorders. When lipid metabolism disorders occur, the intestinal flora in patients also undergo structural changes correspondingly, and the changes in intestinal flora structure can also synchronously affect the changes in blood lipids in patients. At present, Chinese medicine, which has accumulated long-term clinical practice, is gradually becoming a comprehensive treatment for lipid metabolism disorders, and some clinical studies have shown that regulating intestinal flora and balancing intestinal microecology are among the important ways for Chinese medicine to play a role (5). After oral preparation of traditional Chinese medicine (TCM), the unabsorbed components are in contact with intestinal flora, and a series of biotransformations, such as hydrolysis, oxidation, and reduction reactions, occur in the TCM components under the action of flora, making the components to be more easily absorbed and metabolized, and even produce new pharmacological activities. At the same time, the TCM components will have a certain effect on the diversity, richness, and bacterial structure of intestinal flora (6). At present, many studies (7, 8) have suggested that oral preparations of TCMs that regulate intestinal flora can prevent and treat lipid metabolism disorders, but there is no systematic evaluation of this intervention method, and this study searched recent clinical controlled studies to provide more exact evidence for oral preparations of TCMs to prevent and treat lipid metabolism disorders.

## Method

### Criteria for Inclusion of Literature in the Study

#### Literature Type

All studies were randomized controlled trials. We also excluded controlled clinical trials (CCTs), cohort study, case-control study, and case series. Other summaries of experience, reviews, case studies, and studies of heterogeneity were also excluded.

#### Inclusion, Exclusion Criteria

1. Inclusion criteria: The type of literature was domestic and foreign published clinical randomized controlled trials; the participants were adults aged >18 years, with abnormal lipid metabolism, including diabetic patients, obese patients, and patients with ovarian syndrome; the general data of patients such as gender, age, and weight were comparable; The included literature was able to provide sufficient data information for the study to perform calculations.

2. Exclusion criteria: use of probiotic preparations or statins or fibrate lipid-regulating drugs in the trial group; studies with inaccessible full text; studies with incomplete data results; those with duplicate data.

#### Description of Intervention

Since the included studies were randomized controlled studies, two randomized groups were included. The patients in both the groups were given the same basic treatment, aerobic exercise, resistance training, and diet control. The observation group took oral preparations of TCM (the TCM preparations were verified to regulate the number of intestinal flora), while the control group was not given additional measures or only given placebo. The intervention time was more than 3 weeks.

#### Outcome Indicators

In this study, the main outcome indicators included two categories: (a) post-treatment lipid levels, containing TC, triglycerides (TG), and HDL; (b) post-treatment number of intestinal flora, containing enterococcus, enterobacter, lactobacillus, and bifidobacterium.

### Search Strategy

Search databases: CBM, Pubmed, Embase, CNKI, and Wanfang. We also searched relevant literatures by *google scholar* using the keywords “*Traditional Chinese Medicine*” or “*TCM*” or “*Intestinal flora*” or “*gut microbiota*” or “*Metabolic syndrome*.”

### Literature Screening and Data Extraction

Two researchers independently screened the included studies and excluded duplicate articles, and obviously unqualified articles, by reading the titles and abstracts. If there was a conflict of opinion between the two researchers, a third researcher was consulted to resolve the difference of opinion.

Two researchers independently extracted the data, read each included study using a pre-prepared form, and obtained the data from the text, including author, journal name, publication time, number of participants, age, gender composition, weight, body mass index (BMI), disease duration, blood glucose level, four items of blood lipids before intervention, grouping method, number of cases in each group, intervention method, intervention time, and outcome indicators.

If there was no data in the literature for processing, the original author of the literature was contacted to obtain the data. If the data could not be obtained, the literature was discarded.

### Literature Quality Evaluation

We used the Evidence-based Health Care Center of JBI (Joanna Briggs Institute) in Australia (9) to evaluate the evaluation criteria for randomized controlled trials. The evaluation criteria included six aspects: randomization, allocation concealment, blind method, withdrawal and loss to follow-up, ITT (Intention-to-treat) analysis, and baseline comparison. The literatures were divided into three levels: Grade A: meeting the evaluation in the above six aspects, with less possibility of bias; Grade B: partially meeting the evaluation in the above six aspects, with certain possibility of bias; Grade C: most of them did not meet the evaluation, with greater possibility of bias. We only included studies with Grade A or B quality and excluded studies with Grade C quality.

### Statistical Analysis

We used Review Manager 5.3 (released by The Nordic Cochrane Centre, The Cochrane Collaboration, 2014) as an analytical tool in this study. The random effects inverse variance model was calculated, and the effect measure used mean difference (*MD*). We used Cochran *Q* test and *I*^2^ to count the heterogeneity of the study, and *I*^2^ > 50% or *p* < 0.1 indicated statistically significant heterogeneity. The heterogeneity was investigated by piecemeal exclusion method. If the source of heterogeneity was not obtained, only general descriptive analysis was performed. Funnel plots were used to represent publication bias.

## Results

### Literature Search Results

**Figure 1:**
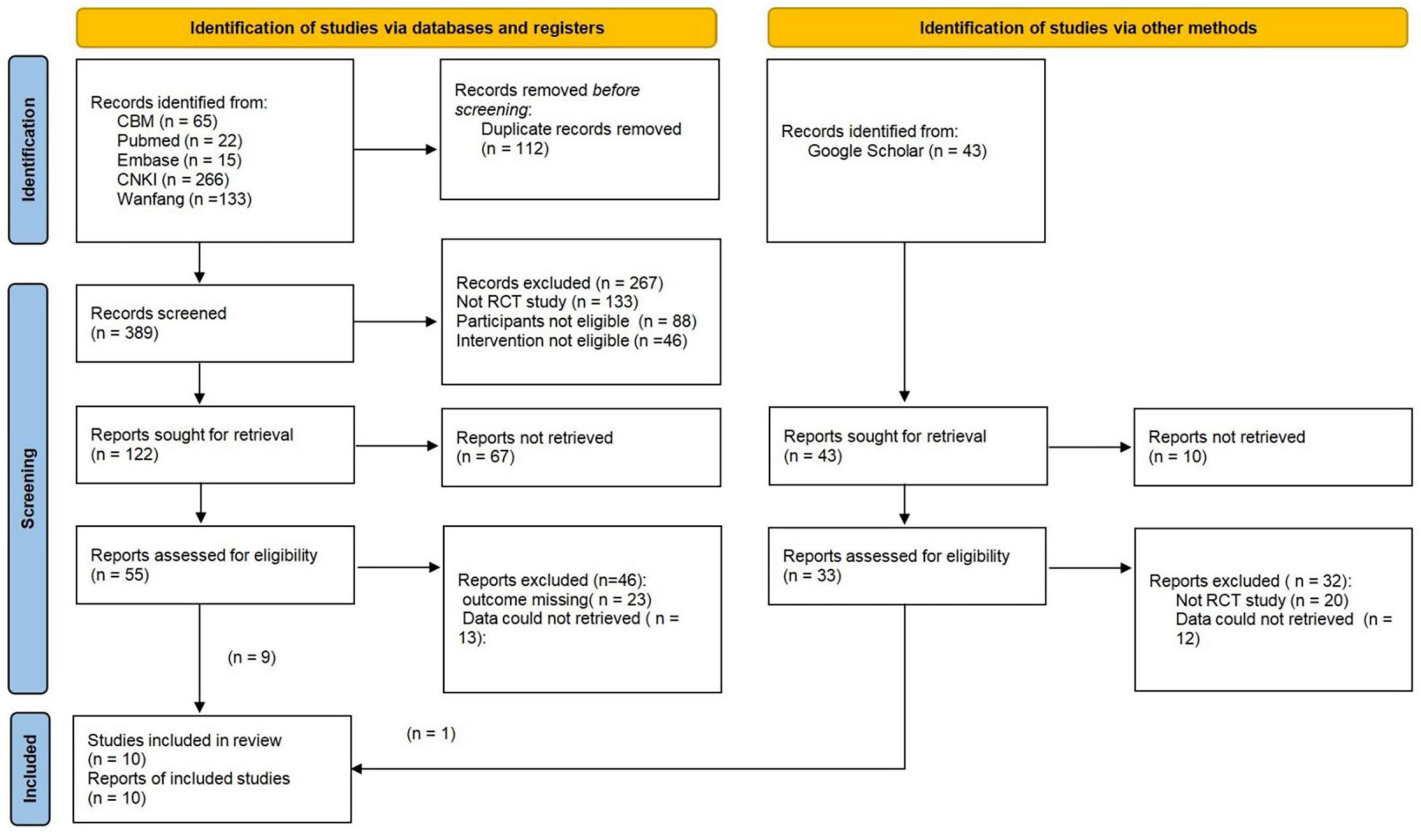
Literature screening flow chart.

Figure 1 shows the results of the literature search and the screening process.

### Basic Characteristics of Included Literatures

A total of 10 articles (10–19) with a total of 835 patients were included in the study and published between 2015 and 2021, with an average intervention time between 1 and 6 months. Specific information is shown in Table 1. Only one study (10) used a single herb, while other studies used a variety of herbal formulas, which are shown in Table 2.

**Table 1:** Basic characteristics of included literatures, characteristics of study subjects, and quality evaluation scores.

**References**	**Patient characteristics**	**Patient age (years)**	**BMI (kg/m^**2**^)**	**Number of cases (E/C)**	**Experimental group intervention**	**Control group intervention**	**Intervention time (Median)**	**Outcome indicators**
Song et al. (9)	Obese female	34.92 ± 6.46	29.99 ± 4.27	13/15	Schisandra chinensis fruit	Placebo	3 mo	(a–c)
Li et al. (10)	Obese patients with ovarian syndrome	20–40	27–29.9	50/50	Chinese herbs for kidney resolving phlegm+ acupuncture	Oral ethinylestradiol cyproterone tablets	3 mo	(a–g)
Chen et al. (11)	Patients with metabolic syndrome	30.5 ± 6.2	32.0 ± 6.58	30/30	Wendan Decoction	Placebo	1 mo	(a–g)
Zhang et al. (12)	Obesity 2 Patients with type 2 diabetes	41–68	19–33	52/54	Shenqi Compound Decoction	Placebo	2 mo	(a–c)
Li et al. (13)	Obese type 2 diabetic patients	51.09 ± 11.34	N/A	49/49	Wenyang Yiqi Huoxue Recipe+Metformin	Metformin	2 mo	(a–g)
Liu et al. (14)	Obese Patients	18–65	>28	24/24	Qi Wei Bai Zhu San	Placebo	3 mo	(b,c)
Du et al. (15)	Patients with glucose resistance	28–70	N/A	20/20	Jiangzhuo Mixture	Placebo	1 mo	(a–c)
Chen et al. (16)	Patients with type 2 diabetes	59.81 ± 5.44	N/A	40/40	Fuzi Lizhong Pills+Glichi	Gleetzil	1 mo	(a–g)
Zhang et al. (17)	Patients with metabolic syndrome	60.3 ± 6.7	28.64 ± 1.69	91/89	Lidan Huatan Huoxue Recipe	Placebo	6 mo	(b,c)
Liu et al. (18)	Spleen deficiency syndrome patients with type 2 diabetes	54.37 ± 6.45	25.01 ± 3.98	48/47	Yiqi Bupi Recipe	Placebo	2 mo	(a–g)

*N/A, Not available; E/C, Experimental/Control group.*

*Outcomes: (a) TC; (b) TG; (c) HDL; (d) number of Enterobacteriaceae; (e) number of Enterococcus; (f) number of Lactobacillus; (g) number of Bifidobacterium*.

**Table 2:** Composition of Chinese herbal formulas.

**Study**	**Name**	**Composition**
Song et al. (9)	Schisandra chinensis	Schisandra chinensis
Li et al. (10)	Chinese herbs for treating kidney and resolving phlegm	Radix Bupleuri, Radix Paeoniae Alba, *Angelica sinensis*, Rhizoma Cyperi, *Fructus aurantii, Ligusticum chuanxiong*, licorice, Atractylodes, tangerine peel, *Poria cocos, Pinellia ternata*, coix seed, *Scutellaria baicalensis*
Chen et al. (11)	Wendan Decoction	Zhuru, *Fructus aurantii, Pinellia ternata*, tangerine peel, *Poria cocos*, roasted licorice, ginger, jujube
Zhang et al. (12)	Shenqi compound decoction	Ginseng, Astragalus membranaceus, *Cornus officinalis*, Chinese yam, *Rehmannia glutinosa, Salvia miltiorrhiza*, cooked rhubarb
Li et al. (13)	Wenyang Yiqi Huoxue Recipe	Rhizoma Aconiti, ginseng, roasted licorice, Atractylodes Macrocephalae, *Poria cocos, Fructus aurantii*, red peony, dogwood meat, bupleurum, Cinnamon Twig, dried ginger, *Salvia miltiorrhiza*
Liu et al. (14)	Qi Wei Bai Zhu San	Radix Pseudostellariae, Atractylodes Macrocephalae, *Poria cocos*, patchouli, wood incense, Pueraria, roasted licorice
Du et al. (15)	Jiangzhuo Mixture	Astragalus membranaceus, *Salvia miltiorrhiza, Atractylodes lancea*, raw coix seed, raw malt, raw lentils, *Gynostemma pentaphyllum*, chicken nuggets, *Pueraria lobata*
Chen et al. (16)	Fuzi Lizhong Pills	Aconite, *Astragalus membranaceus, Pinellia ternata*, ginseng, roasted licorice, tangerine peel, *Atractylodes macrocephala*, huangcen and dried ginger
Zhang et al. (17)	Lidan Huatan Huoxue Recipe	Corn whisker, *Polygonum cuspidatum, Pinellia ternata, Citrus sinensis, Salvia miltiorrhiza*
Liu et al. (18)	Yiqi Bupi Recipe	*Astragalus membranaceus, Codonopsis pilosula, Atractylodes macrocephala, Poria cocos*, licorice

### Risk of Bias Analysis and Quality Assessment

In this study, all literatures were randomized controlled trials, with descriptive blind method (single-blind or double-blind), but only literatures (10) had descriptive allocation hide, only literatures (14) recorded drop-out cases in detail, all literatures used ITT analysis, with descriptive baseline comparison.

1. Randomized allocation method: all literature in this study were randomized controlled trials.
2. Allocation concealment scheme: literature (10) used descriptive allocation concealment, and the allocation concealment scheme of the remaining 9 literatures was not described.
3. Blinding: all literature used descriptive blinding (single or double-blind).
4. The literature (14) recorded the withdrawal cases in detail, and the other literature studies had no withdrawal or lost visits.
5. ITT analysis: All literature used ITT analysis.
6. Baseline comparisons: All literature had descriptive baseline comparisons. The final assessment was that the overall quality of the literature was good. See Table 3.

**Table 3:** Methodological quality assessment and risk of bias analysis based on JBI (Joanna Briggs Institute).

**Study**	**Random sequence generation**	**Classification hiding**	**Blind method**	**Withdrawal and Lost to Follow-up**	**ITT analysis**	**Baseline comparison**	**Quality level**
Song et al. (9)	Adopt	Described	Double-blind	Not described	Adopt	Described	Grade A
Li et al. (10)	Adopt	Not described	Single-blind	Not described	Adopt	Described	Grade B
Chen et al. (11)	Adopt	Not described	Single-blind	Not described	Adopt	Described	Grade B
Zhang et al. (12)	Adopt	Not described	Single-blind	Not described	Adopt	Described	Grade B
Li et al. (13)	Adopt	Not described	Single-blind	Described	Adopt	Described	Grade A
Liu et al. (14)	Adopt	Not described	Double-blind	Not described	Adopt	Described	Grade B
Du et al. (15)	Adopt	Not described	Single-blind	Not described	Adopt	Described	Grade B
Chen et al. (16)	Adopt	Not described	Single-blind	Not described	Adopt	Described	Grade B
Zhang et al. (17)	Adopt	Not described	Single-blind	Not described	Adopt	Described	Grade B
Liu et al. (18)	Adopt	Not described	Single-blind	Not described	Adopt	Described	Grade B

### Meta-Analysis Results

**(A) Total cholesterol (mmol/l)**

**Figure 2:**
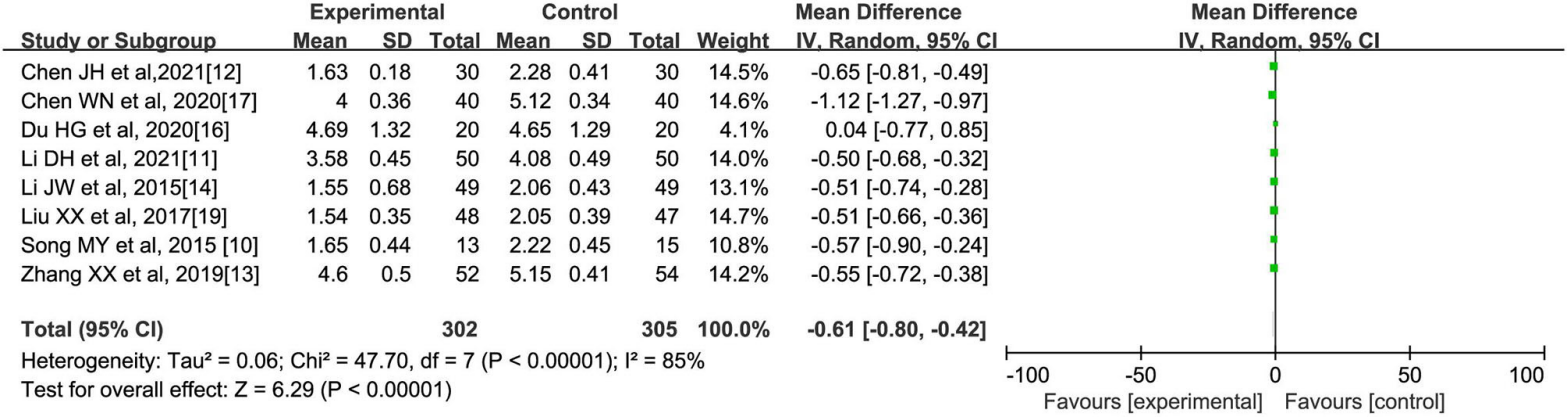
Effect of oral Chinese herbs on total cholesterol (TC) in patients with dyslipidemia.

A total of eight literatures (10–14, 16, 17, 19) reported the comparison of TC indicators of patients after oral Chinese medicine intervention, with 302 cases in the experimental group and 305 cases in the control group, and the internal heterogeneity of the eight literatures was statistically significant (*I*^2^ = 85%, *p* < 0.00001). Using the random-effects model, it was shown that Chinese medicine intervention could reduce the TC level of patients with abnormal lipid metabolism [*MD* = −0.61, 95% confidence interval (95% CI) (−0.80, −0.42), *Z* = 6.29, *p* < 0.00001], as shown in Figure 2.

**(B) Triacylglycerol (mmol/l)**

**Figure 3:**
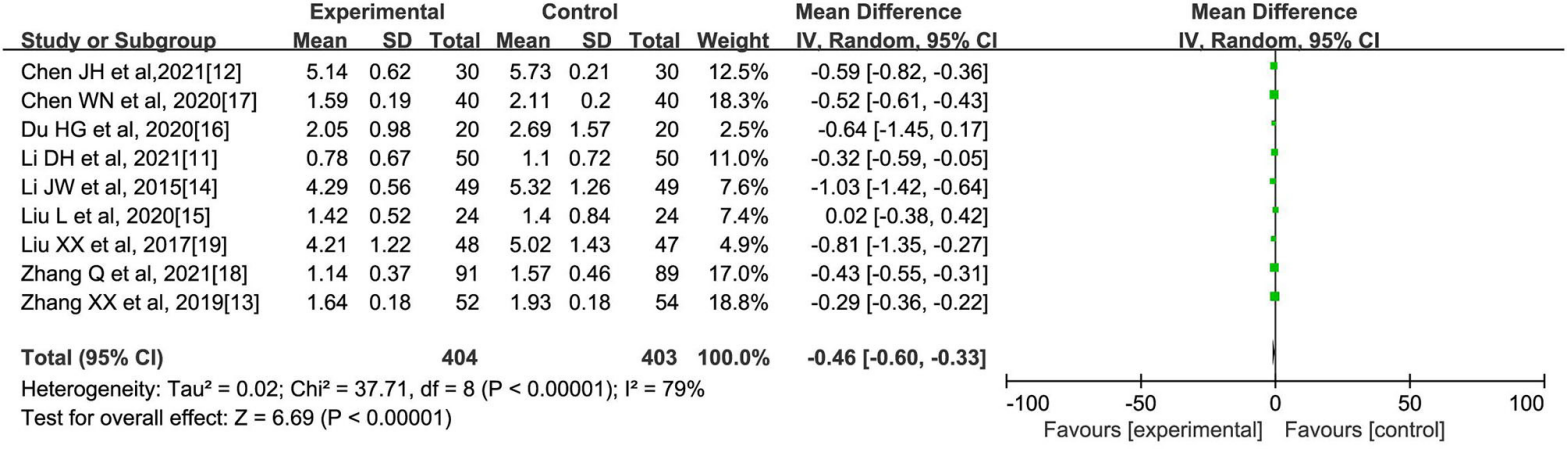
Effect of oral Chinese herbs on triglycerides (TG) in patients with dyslipidemia.

A total of 9 literatures (11–17) reported the comparison of TG indicators after oral Chinese medicine intervention, with 404 cases in the experimental group and 403 cases in the control group. The internal heterogeneity of the nine literatures was statistically significant (*I*^2^ = 79%, *p* < 0.00001). The random-effects model was used to obtain that Chinese medicine intervention could reduce the triacylglycerol level in patients with abnormal lipid metabolism [*MD* = −0.46, 95% CI (−0.60, −0.33), *Z* = 6.69, *p* < 0.00001], as shown in Figure 3.

**(C) High-Density lipoprotein (mmol/l)**

**Figure 4:**
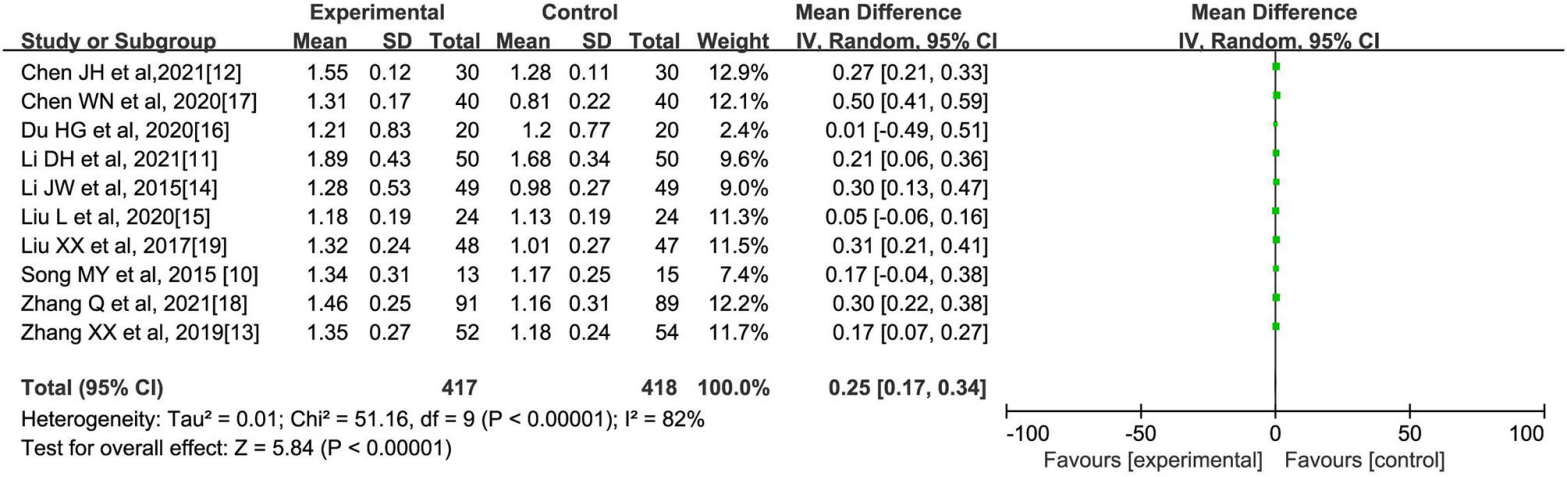
Effect of oral Chinese herbs on high-density lipoprotein (HDL) in patients with dyslipidemia.

All literatures (10–19) reported the comparison of HDL indicators after oral Chinese medicine intervention, with a total of 417 cases in the experimental group and 418 cases in the control group. The internal heterogeneity of the 10 literatures was statistically significant (*I*^2^ = 82%, *p* < 0.00001). The random-effects model was used to obtain that Chinese medicine intervention could increase the HDL level in patients with abnormal lipid metabolism [*MD* = 0.25, 95% CI (0.17, 0.34), *Z* = 5.84, *p* < 0.00001], as shown in Figure 4.

**(D) Number of Enterobacteriaceae**

**Figure 5:**
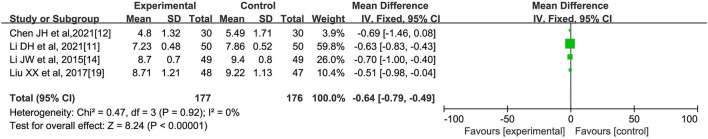
Effect of oral Chinese medicine on the number of intestinal enterobacteria in patients with dyslipidemia.

A total of four literatures (11, 12, 14, 19) reported the comparison of the number of intestinal enterobacteria in patients after oral Chinese medicine intervention, with 177 cases in the experimental group and 176 cases in the control group. The four literatures had no heterogeneity (*I*^2^ = 0%, *p* = 0.92). Using the fixed-effect model, it was obtained that Chinese medicine intervention could reduce the number of enterobacteria in patients with abnormal lipid metabolism [*MD* = −0.64, 95% CI (−0.79, −0.49), *Z* = 8.24, *p* < 0.00001], as shown in Figure 5.

**(E) Number of Enterococci**

**Figure 6:**
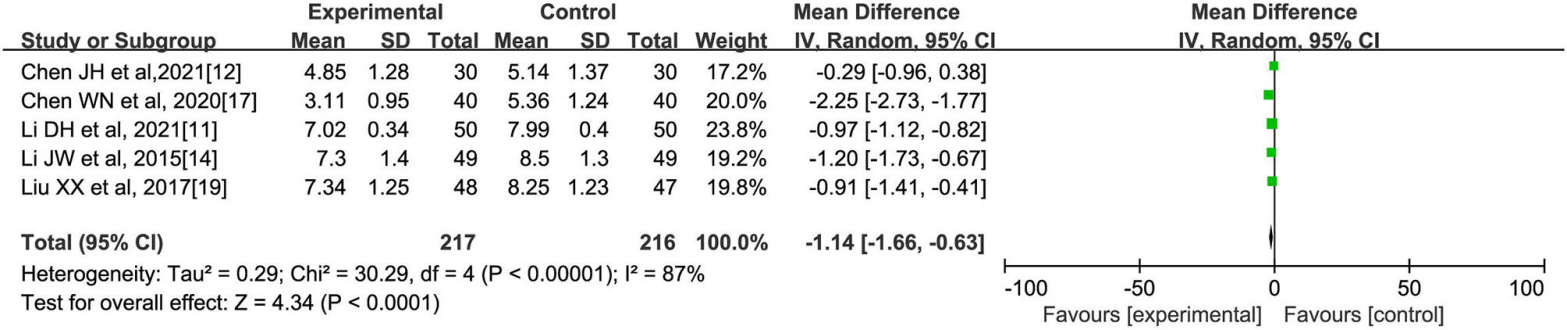
Effect of oral Chinese herbs on the number of intestinal enterococci in patients with lipid abnormalities.

A total of five literatures (11, 12, 14, 17, 19) reported the comparison of the intestinal cocci counts in patients after oral herbal intervention, with a total of 217 cases in the experimental group and 216 cases in the control group. The analysis showed significant heterogeneity among the five studies (I2 = 87%, *p* < 0.00001). Meta-analysis using random-effects model showed that the number of enterococci in the experimental group was significantly lower than that in the control group [MD=-1.14, 95% CI (−1.66, −0.63), *Z* = 4.34, *p* < 0.00001], which suggested that herbal interventions could reduce the number of enterococci in patients with abnormal lipid metabolism, as shown in Figure 6.

**(F) Number of Lactobacillus**

**Figure 7:**
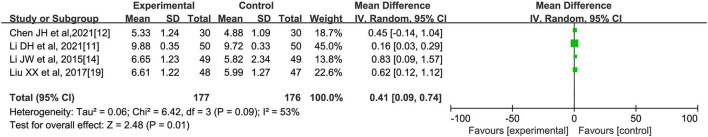
Effect of oral Chinese herbs on the number of lactobacillus in patients with lipid abnormalities.

A total of four literatures (11, 12, 14, 19) reported a comparison of intestinal lactic acid bacteria counts in patients after oral herbal interventions, with a total of 177 cases in the experimental group and 176 cases in the control group. The analysis showed significant heterogeneity among the four studies (*I*^2^ = 53%, *p* = 0.09). Meta-analysis using a random-effects model showed that the number of intestinal lactobacilli in the experimental group was significantly higher than that in the control group [*MD* = 0.41, 95% CI (0.09, 0.74), *Z* = 2.48, *p* = 0.01], which suggested that the herbal intervention could increase the number of intestinal lactobacilli in patients with abnormal lipid metabolism, as shown in Figure 7.

**(G) Number of Bifidobacteria**

**Figure 8:**
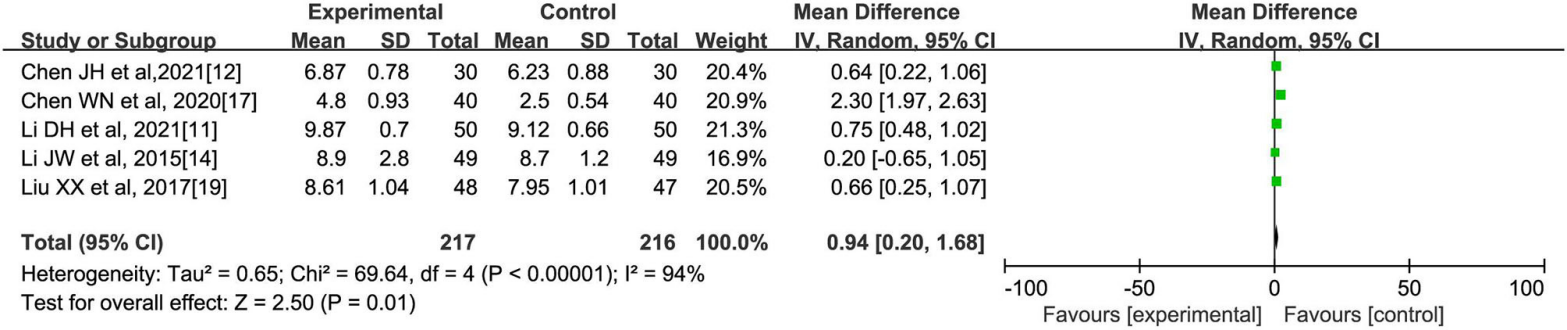
Effect of oral Chinese herbs on the number of intestinal bifidobacteria in patients with lipid abnormalities.

A total of five literatures (11, 12, 14, 17, 19) reported a comparison of intestinal bifidobacteria counts in patients after oral herbal interventions, with a total of 217 cases in the experimental group and 216 cases in the control group. The analysis showed significant heterogeneity among the five studies (*I*^2^ = 94%, *p* < 0.00001). Meta-analysis using a random effects model showed that the number of intestinal bifidobacteria in the experimental group was significantly higher than that in the control group [*MD* = 0.94, 95% CI (0.20, 1.68), *Z* = 2.50, *p* = 0.01], which suggests that herbal interventions can increase the number of intestinal bifidobacteria in patients with abnormal lipid metabolism, as shown in Figure 8.

### Heterogeneity Survey and Sensitivity Analysis

In the analysis of the effect of oral Chinese herbs on TC levels in patients, we adopted a case-by-case exclusion method to find the source of heterogeneity. However, after excluding any article, the remaining articles still had heterogeneity, and the heterogeneity between articles came from multiple aspects and may be related to factors such as different characteristics of patients, age, and different formulations of Chinese herbs.

### Publication Bias Analysis

**Figure 9:**
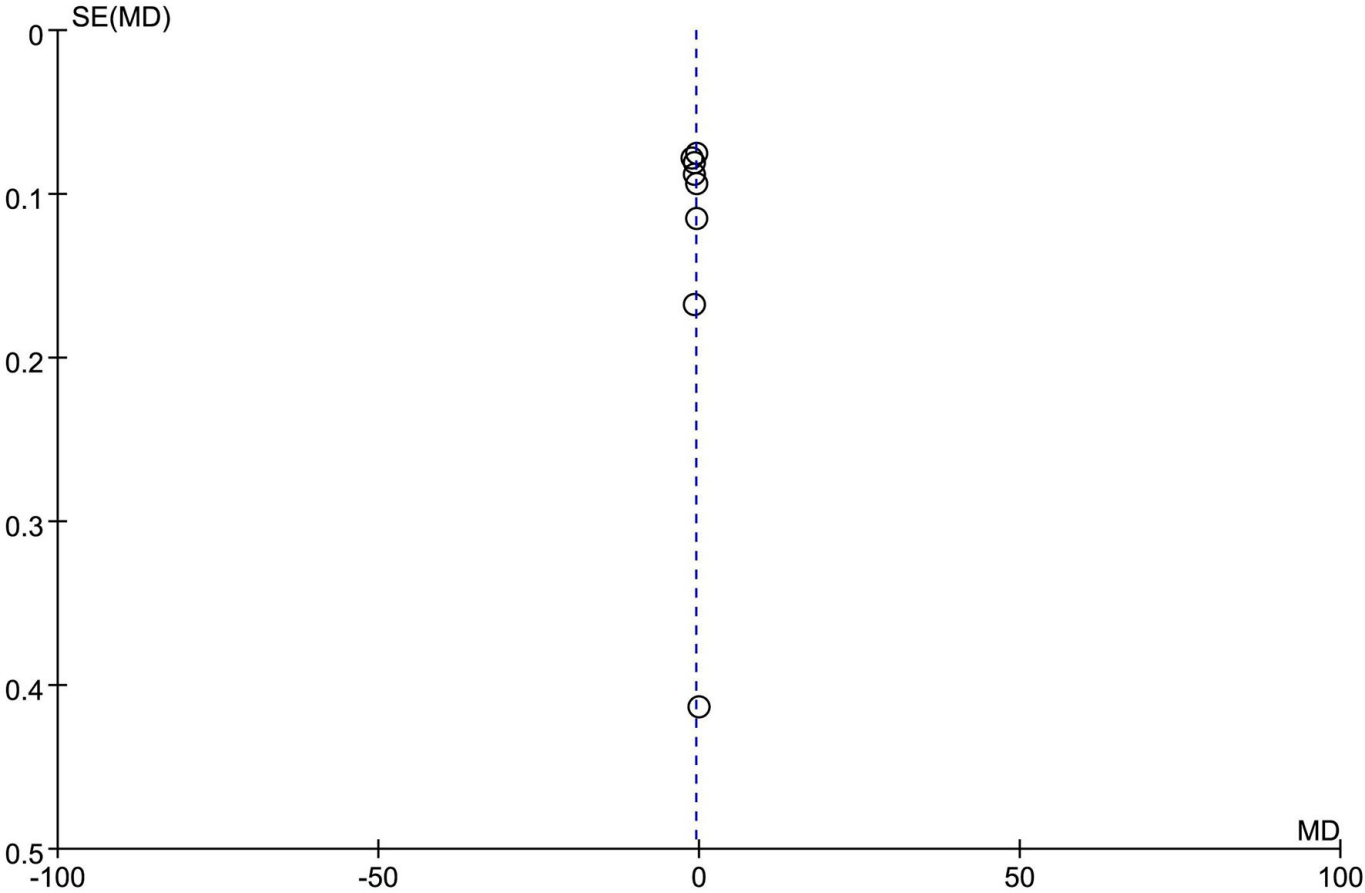
Funnel plot analysis.

In the analysis of the effect of oral Chinese herbs on TG levels in patients, a funnel plot was drawn, and the left and right distributions of the 10 included articles were symmetrical, suggesting that there was no publication bias, as shown in Figure 9.

## Discussion

Lipid metabolism is regulated by genetics, neurohumoral, hormones, enzymes and tissues, and organs such as the liver. When these factors are abnormal, they can cause lipid metabolic disorders and pathophysiological changes in the related organs, hyperlipoproteinemia, lipidosis, and the resulting clinical syndromes, such as obesity, ketoacidosis, fatty liver, and neonatal scleredema (20). Statins are commonly used drugs for the treatment of dyslipidemia, but such drugs may bring many adverse effects, such as liver and kidney dysfunction, abnormal neuromuscular responses, increased risk of diabetes, and cognitive decline (21). Accumulating evidence suggests that gut flora structure is closely related to the development of lipid metabolism disorders (22). Intestinal flora are a unique microecosystem that depend on the host and affect each other, and when the structure of intestinal flora is disturbed, the dynamic ecological balance of the intestine is disrupted, which can affect bile acid metabolism, thereby changing lipid metabolism, which leads to dyslipidemia (23). Studies have shown (24) that TCM can indirectly regulate blood lipids by regulating intestinal flora. The polysaccharide components in Chinese medicine can promote the proliferation of probiotics such as Bifidobacterium, Lactobacillus, and Haemophilus, and also indirectly inhibit the growth of harmful bacteria (24).

In this meta-analysis, 10 controlled clinical studies on TCMs that can regulate intestinal flora, for the prevention and treatment of dyslipidemia were included, and the results showed that after the intervention of TCM, the TC and triacylglycerol levels of the patients decreased compared with the control group, while the HDL level increased, which suggested that such TCM could contribute to the recovery of blood lipids in patients with abnormal lipid metabolism. On the other hand, after the intervention of TCM, the number of harmful bacteria in the intestinal flora of the patients decreased, and the number of beneficial bacteria such as lactobacillus and bifidobacterium increased, which indicated that the intervention of TCM helped the patients to restore the microecological balance in the intestine. The hydrolysis, oxidation, and reduction reactions of TCM components occur under the action of intestinal flora, and hence TCM components are more likely to absorb, metabolize, and even produce new active substances, and the efficacy of TCM will show enhanced activity, while intestinal flora will also be affected by TCM while biotransforming TCM, and the structure of intestinal flora will change under the action of TCM (25). Various herbal medicines have been found to be involved in this bacterial regulation, and studies (26) have shown that *Codonopsis pilosula* can increase the level of lactobacillus in the intestine of mice, while inhibiting *Escherichia coli*. *Atractylodes macrocephala* Koidz, *Astragalus membranaceus, Lycium barbarum*, and *Rehmannia glutinosa* can promote the proliferation of bifidobacteria, lactobacillus, *Lactobacillus acidophilus*, and other beneficial flora, and inhibit the growth of the large intestine (27).

In this study, the heterogeneity sources were investigated by excluding one by one. However, after excluding each article, the heterogeneity existed in the remaining literatures, which indicated that multiple aspects of heterogeneity sources might be related to different characteristics of patients, age, different formulations of TCMs and other factors. In this study, JBI's scale was used to evaluate the quality of the 10 included articles. The scores showed that the quality of the articles was good, but most of the articles did not describe the dropout cases, which may have a large implementation bias. Publication bias analysis showed a uniform distribution on both sides, suggesting the absence of publication bias. In future studies, more literatures with less heterogeneity and high quality can be selected for further analysis.

## Summary

In summary, the application of oral preparations of TCMs that regulate intestinal flora in the prevention and treatment of lipid metabolism disorders can increase the colonization of beneficial bacteria in the intestine of patients, inhibit the growth of harmful bacteria, and restore the intestinal microecological balance, thus indirectly acting on the blood lipid regulation of patients and contributing to the recovery of dyslipidemia. However, based on the heterogeneity and publication bias in the studies, the topic still needs to be further explored by including more controlled clinical studies with better quality in clinical practice.

## Data Availability Statement

The original contributions presented in the study are included in the article/supplementary material, further inquiries can be directed to the corresponding authors.

## Ethics Statement

The studies involving human participants were reviewed and approved by this study was approved by the Ethics Committee of our hospital. The patients/participants provided their written informed consent to participate in this study.

## Author Contributions

WG and WZ are the mainly responsible for the writing of the article. CC is mainly responsible for research design. WG is mainly responsible for data analysis. WZ and CC are responsible for the guidance of the entire research. The corresponding author is WG and she is responsible for ensuring that the descriptions are accurate and agreed by all authors. All authors may have contributed in multiple roles. All authors contributed to the article and approved the submitted version.

## Conflict of Interest

The authors declare that the research was conducted in the absence of any commercial or financial relationships that could be construed as a potential conflict of interest.

## Publisher's Note

All claims expressed in this article are solely those of the authors and do not necessarily represent those of their affiliated organizations, or those of the publisher, the editors and the reviewers. Any product that may be evaluated in this article, or claim that may be made by its manufacturer, is not guaranteed or endorsed by the publisher.
